# The Bioinformatic Applications of Hi-C and Linked Reads

**DOI:** 10.1093/gpbjnl/qzae048

**Published:** 2024-06-21

**Authors:** Libo Jiang, Michael A Quail, Jack Fraser-Govil, Haipeng Wang, Xuequn Shi, Karen Oliver, Esther Mellado Gomez, Fengtang Yang, Zemin Ning

**Affiliations:** School of Life Sciences and Medicine, Shandong University of Technology, Zibo 255049, China; The Wellcome Sanger Institute, Wellcome Genome Campus, Hinxton, Cambridge CB10 1SA, UK; The Wellcome Sanger Institute, Wellcome Genome Campus, Hinxton, Cambridge CB10 1SA, UK; School of Life Sciences and Medicine, Shandong University of Technology, Zibo 255049, China; College of Food Science and Technology, Hainan University, Haikou 570228, China; The Wellcome Sanger Institute, Wellcome Genome Campus, Hinxton, Cambridge CB10 1SA, UK; The Wellcome Sanger Institute, Wellcome Genome Campus, Hinxton, Cambridge CB10 1SA, UK; School of Life Sciences and Medicine, Shandong University of Technology, Zibo 255049, China; The Wellcome Sanger Institute, Wellcome Genome Campus, Hinxton, Cambridge CB10 1SA, UK

**Keywords:** Long-range NGS reads, Hi-C, Linked Reads, Genome assembly, Quality assessment

## Abstract

Long-range sequencing grants insight into additional genetic information beyond what can be accessed by both short reads and modern long-read technology. Several new sequencing technologies, such as “Hi-C” and “Linked Reads”, produce long-range datasets for high-throughput and high-resolution genome analyses, which are rapidly advancing the field of genome assembly, genome scaffolding, and more comprehensive variant identification. In this review, we focused on five major long-range sequencing technologies: high-throughput chromosome conformation capture (Hi-C), 10X Genomics Linked Reads, haplotagging, transposase enzyme linked long-read sequencing (TELL-seq), and single- tube long fragment read (stLFR). We detailed the mechanisms and data products of the five platforms and their important applications, evaluated the quality of sequencing data from different platforms, and discussed the currently available bioinformatics tools. This work will benefit the selection of appropriate long-range technology for specific biological studies.

## Introduction

Next-generation sequencing (NGS) technologies have revolutionized the field of genomics and genetics, providing low-cost and high-throughput data at an unprecedented scale. However, most NGS technologies make an underlying assumption that all relevant genetic information can be reconstructed from smaller fragments: short (100–250 bp) or long (> 10,000 bp) reads. Such reads are “short range” or “local”, because they contain only information about the bases within the read, in contrast to “long-range”, “non-local”, or “linked” reads, which retain additional contextual information about the spatial position of the read within the complex, 3-dimensional (3D) physical structure of the DNA within and between chromosomes.

We emphasize that, despite the similar terminology, long-range reads are conceptually distinct from long reads. In terms of sequence format, long reads are contiguous, while long-range reads are non-contiguous with sequences separated by large gaps. In terms of span distances, long-range reads are much longer, *e.g.*, high-throughput chromosome conformation capture (Hi-C) reads can even span over a whole chromosome. In conclusion, long-range reads provide additional non-local information, and can take the form of either short or long reads. As a matter of fact, most long-range technologies currently use short reads.

Without the additional context of non-local information, it remains challenging to reliably identify structural variations (SVs) with short reads. Although short reads can be used to identify SVs at base-pair resolution, utilizing only short-range information suffers from a higher false discovery rates than long reads [1]. It is also difficult to phase millions of short reads to achieve a haplotype-resolved genome, particularly for highly repetitive sequences, complex heterozygosity, and large polyploid genomes [2,3].

Local long reads can sidestep many of the issues with local short reads since, although they contain only local information, their large size makes it much easier to uniquely localize them within the genome [4,5]. Currently, two major long-read technologies, Pacific Biosciences (PacBio) single-molecule real-time (SMRT) sequencing and Oxford Nanopore Technologies (ONT) nanopore sequencing, are used for long-read-based genome analysis [4]. However, the long-read method has two drawbacks: (1) higher costs and lower throughput, and (2) higher DNA input requirements compared to short-read sequencing. These limitations increase the cost threshold for a long-read approach when trying to resolve structural rearrangements.

It might seem that the advent of long reads has made long-range platforms into niche endeavours, with large projects such as the Vertebrate Genomes Project (VGP) [6], the Darwin Tree of Life (DToL; https://www.darwintreeoflife.org), and the Earth BioGenome Project (EBP) [6] using long-read platforms to achieve their ambitious goals. However, the throughput and cost issues associated with long reads means that using them as the sole means of long-range information in the *de novo* assembly of tens of thousands of genomes will likely be prohibitive. This is especially true if chromosome-level assemblies are desired, since long reads are still much shorter than chromosomes and hence do not provide chromosome-scale context. Therefore, a more cost-effective method for inferring long-range information is needed. Whilst long-range information alone has fallen behind long reads in *de novo* assembly, it can serve as a cheap and fast supplement to long reads, forming a strong hybrid approach that token by several large projects (including VGP and DToL) in fact. Long-range platforms are far from defunct.

Several technologies for storing long-range information in the form of short reads have been developed to reconstruct a complete long-chain DNA molecule, the most prominent of which are the “Hi-C” and “Linked Reads” technologies [7]. 10X Genomics provides perhaps the best Linked Read strategy, capable of generating long-range information from standard approaches based on short reads [8]. In recent years, 10X Genomics Linked Reads (henceforth simply as “10X”) technology has been widely used, but a variety of other barcode-based methods, such as TruSeq, single-tube long fragment read (stLFR), transposase enzyme linked long-read sequencing (TELL-seq), LoopSeq, and haplotagging, have been developed for ultralow DNA input, high per-base resolution, and low costs [9–12].

In this study, we focused on five major long-range sequencing technologies, *i.e.*, Hi-C, 10X, haplotagging, TELL-seq, and stLFR. First, we detailed the protocols and mechanisms of the five platforms. Second, we proposed some criteria to evaluate the quality of sequencing data from different platforms, and applied these criteria to discuss the characteristics of datasets either downloaded from public resources or sequenced by us in this project. Third, we reviewed the practical applications of these technologies in efforts such as genome scaffolding, *de novo* assemblies, and variation screens. Finally, we discussed the strengths and weaknesses of a number of software tools which are commonly used for genome analysis with long-range reads.

## Platforms

In this section, we briefly detailed the five platforms of interest: Hi-C, 10X, haplotgging, TELL-seq, and stLFR, focusing on the protocols used to generate the long-range data and how this long-range information is manifested in the data products. Across these five platforms, long-range, non-local information is stored in one of two ways: either in Hi-C reads, in which two reads are coupled together to indicate a relationship between them; or Linked Reads, in which reads are tagged or labeled in some way to indicate their origin. This qualitative difference on the non-local information provided by the platform helps determine which platform is most suitable for a given application.

### Hi-C

**Figure 1:**
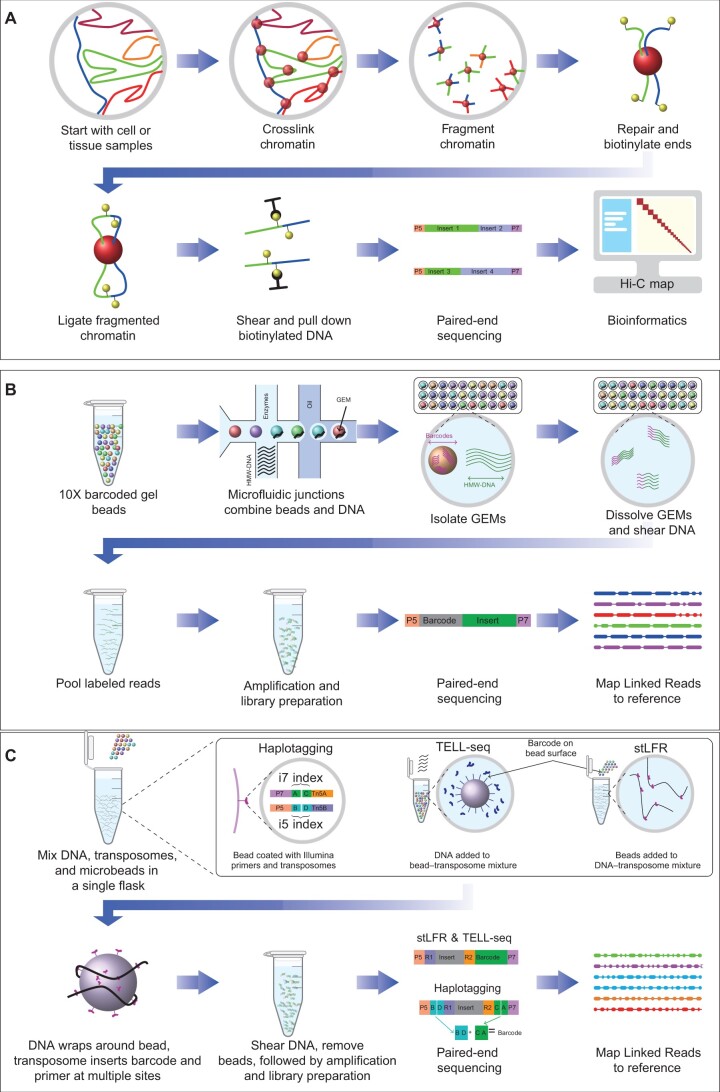
Work flow of library preparation for five long-range platforms **A**. Hi-C. **B**. 10X Genomics Linked Reads. **C**. Haplotagging, TELL-seq, and stLFR. Hi-C, high-throughput chromosome conformation capture; TELL-seq, transposase enzyme linked long-read sequencing; stLFR, single-tube long fragment read; GEM, gel bead in emulsion; HMW-DNA, high molecular weight DNA.

Hi-C is the culmination of several generations of chromosome conformation capture technologies, which probes the spatial organization of chromatin within a cell at a genome-wide scale [13]. Since chromatin is a complex 3D structure, this information allows researchers to detect long-range interactions between segments within a chromosome or between different chromosomes. This feature enables Hi-C to improve *de novo* assembly and phase heterozygous genome variants onto haplotypes, since homologous chromosomes tend to occupy distinct territories within the nuclei [14], resulting in spatially distinct sequences associated with each haplotype. Hi-C technology follows the protocol shown in **Figure 1**A. (1) The nuclear chromatin is crosslinked using formaldehyde. By design, these crosslinks occur preferentially between strands that are close in 3D space. (2) Crosslinked chromatin is cut using a restriction enzyme. (3) The ends of crosslinked segments are repaired by filling in with biotin-labeled nucleotides. (4) DNA ligase is used to cyclize the blunt-ends, followed by the degradation of the proteins that bind the DNA fragments. (5) The circular crosslinked fragments are randomly sheared again using sonication or other methods, and then the biotin-labeled DNA fragments are captured with streptavidin-conjugated beads and amplified before sequencing.

The final Hi-C data consist of a large number of “deliberately chimeric” paired short reads, each originating exclusively from two crosslinked fibers that can be located very far away from each other in the linear genome or even on different chromosomes entirely. The generated paired reads are then mapped to a genome assembly and used to create a high-resolution interaction map within and between chromosomes. Aeras with a large number of paired chimeric reads are then inferred to be chromain regions in spatial proximity.

### 10X

10X is a product formerly provided by 10X Genomics. In 10X sequencing, long-range information is retained by combining 5′ barcoding with standard short-read sequencing [15,16], producing short Linked Reads (where we are making a distinction between Linked Reads as a genomic technique, and the 10X “Linked Reads” products produced by this technique) with a “memory” of the larger-scale locality that they were derived from, and hence making it easier to assemble the resulting dataset. The resulting reads have the potential to improve the quality of genome assembly and expand the range of linking information along the chromosome to define haplotypes. The 10X protocol (Figure 1B) is as follows. (1) First, DNA is prepared and sheared into long DNA fragments (ideally > 100 kb). (2) At two microfluidic junctions, the high molecular weight DNA (HMW-DNA) is then combined with enzymes and gel beads loaded with random primers and barcode sequences in an oil–surfactant solution to form “gel beads in emulsion” (GEMs). Each GEM captures around 10 HMW-DNA fragments. (3) The GEMs are then isolated in partitions and the beads are dissolved, releasing the barcodes and primers uniquely to the HMW-DNA fragments in that partition. (4) Each partition is then amplified, sheared, and put through standard library preparation methods for sequencing — in this case, by Illumina paired-end sequencing.

The 10X process generates a number of short reads prepended by a unique barcode identifying the GEM that they originated from, *i.e.*, all reads sharing the same barcode are defined as Linked Reads. Some of these Linked Reads might be spuriously linked by one bead capturing multiple fragments, giving them all the same barcode (a “barcode collision”); however, the key statistic is that since each GEM captures only a few HMW-DNA fragments, the probability that a second fragment which shares the same barcode also originates from a nearby location in the genome is very low. Therefore, it is relatively easy to distinguish the spurious links, and so the barcode acts to (nearly) uniquely group sets of reads together as being spatially co-located. This, for example, makes it much easier to phase short reads as the entire barcoded molecule must be simultaneously phased.

Although 10X sequencing can reconstruct multi-megabase phase blocks by assembling short reads with barcode information, it still has some drawbacks, such as relatively high costs in library preparation and counterintuitively decreased performance when faced with smaller genomes. This is because the 10X platform is optimized for the human genome; the partitions adapted for the smaller genome size become saturated at high frequency, leading to the statistics that favour “barcode collisions” in non-human cases.

Significantly, this product was withdrawn and discontinued in 2020 (https://www.10xgenomics.com/products/linked-reads). However, we still included this platform in our analysis to maintain continuity with previous benchmarking and comparison efforts, and since future 10X Genomics products may be comparable to this previous iteration. The 10X human genome reads used in this review were produced by Genome in a Bottle (GIAB) and can be downloaded from ftp://ftp-trace.ncbi.nlm.nih.gov/ReferenceSamples/giab/data/NA12878/10Xgenomics_ChromiumGenome_LongRanger2.0_06202016/NA12878_GRCh38.bam. The 10X hummingbird dataset has been archived on National Center for Biotechnology Information (NCBI)/ EMBL’s European Bioinformatics Institute (EMBL-EBI) (BioProject: PRJNA489243).

### Haplotagging

Several other technologies have been developed to provide an alternative form of Linked Reads in the absence of 10X. Meier et al. [12] developed “haplotagging”, a simple and relatively low-cost Linked Read sequencing technique, which allows high-throughput sequencing without losing haplotype information while maintaining the power, accuracy, and scalability of standard Illumina sequencing.

Haplotagging is a transposon bead-based technology that employs transposomes containing bead-specific barcoded adaptors. These technologies utilize the tendency of HMW-DNA segments to wrap around microbeads, providing many points of contact between the bead and the DNA. The full protocol is as follows (Figure 1C). (1) As in the 10X protocol, HMW-DNA (ideally > 100 kb) is prepared. (2) The HMW-DNA is mixed with the barcoded beads. Each bead carries a standard Illumina Nextera Tn5 transposon adaptor, augmented with 1 of 85 million barcodes, and captures only a single DNA fragment. (3) Transposition transfers the barcoded adaptors into the long DNA fragments, followed by polymerase chain reaction (PCR) amplification to generate a sequencing-ready library. (4) Finally, the libraries are sequenced using an Illumina platform.

The result is that the initial HMW-DNA fragments are broken into smaller units, with all containing a unique barcode, which can be sequenced on short-read sequencers. Subsequently, all the reads originating from the same HMW-DNA fragment can be grouped by their barcode and correctly mapped to that fragment. The key difference between haplotagging technology and 10X is that DNA molecules tend to interact only with a single bead in haplotagging, instead of ∼ 10 fragments per bead (for humans) in 10X. In addition, each bead is tagged with four barcode fragments that are distributed in the standard i5/7 index positions of the Illumina Nextera adaptor design. Thus, library preparation and barcoding are performed simultenously within the same tube using standard molecular biology equipment, making the process both cheaper (the original work claims a 99% cost reduction) and easier. The data output is very similar to that of the 10X platform: a series of short reads prepended by a barcode, indicating which reads originated from a similar vicinity. Since the fragment–bead interaction is close to 1:1 in haplotagging, instead of ∼ 10:1 in 10X, each fragment is genuinely uniquely barcoded, resulting in fewer barcode collisions, as demonstrated in Figures S1 and S2. In addition, the 4-fragment nature of the barcode is designed to allow for error-correction in the barcode read, enabling more robust barcode identification. However, the fragments are prone to display PCR duplication errors [17], and the product is not yet at the stage of commercial deployment.

### TELL-seq

TELL-seq is another Linked Read sequencing technology that is very similar to the haplotagging platform. However, it is currently commercially available through Sage Science.

We note that the name of TELL-seq (*i.e.*, transposase enzyme linked long-read sequencing) falls afoul of the terminology confusion referenced earlier. Under the terminology that we have enforced, the Linked Reads produced are long-range but not long-read.

The workflow of TELL-seq technology is as follows (Figure 1C). (1) Genomic DNA (0.1–5 ng), barcoded TELL beads (3–10 million), and transpososomes are mixed in a PCR tube. (2) The transpososomes and DNA segments interact to form strand transfer complexes (STCs), which are connected to the barcode sequence on the TELL bead surface. (3) The transposase is removed, the DNA fragment is cut into two parts in the STC, and the beads are removed, leaving a DNA fragment connected to a transposon, which is in turn connected to a barcode. (4) The barcoded DNA molecules are amplified with P5 and P7 adaptors before Illumina sequencing.

The library preparation for TELL-seq differs from haplotagging in mostly minor ways, with the primary distinction being in the form that the barcode takes: TELL-seq uses simpler 18-bp barcodes, rather than the 24-bp barcodes for haplotagging. This allows for a larger number of unique barcodes which reduces the probability of a collision, but lacks the error-correction feature.

### stLFR

stLFR is another Linked Read technology developed by MGI, which provides long-range sequencing information using short reads [18], similar to other Linked Read methods discussed. The stLFR platform differs from TELL-seq and 10X in terms of its transposases, barcode system, and barcoding technique used during library preparation. The workflow of stLFR is as follows (Figure 1C). (1) Long DNA molecules are treated with Tn5 enzyme to generate transposon-inserted DNA in a PCR tube. (2) Hybridization buffer is added, and 2.8-µm clone barcode beads are mixed into the PCR tube. (3) Under optimized temperature and buffer conditions, the transposon-inserted DNA is captured by beads via the hybridization sequence, after which the transposons are ligated to the barcode adapters. (4) Transposases are removed, and excess oligos are digested before the second adapters are ligated onto barcoded DNA molecules. (5) These barcoded DNA molecules undergo PCR and cyclization before the co-barcoded subfragments can be sequenced on BGISEQ-500 or an equivalent platform.

Like haplotagging, an important feature of stLFR is its ability to achieve near single-molecule co-barcoding by utilizing a large excess of microbeads and approximately 3.6 billion unique barcode sequences, thereby reducing the barcode collision rate. Additionally, the ability to perform all reactions within a PCR tube makes it easy to scale up for multiple samples and automation purposes. The stLFR reads produced by MGI were downloaded from the GIAB website: https://ftp-trace.ncbi.nlm.nih.gov/giab/ftp/data/AshkenazimTrio/HG002_NA24385_son/stLFR/stLFR_NA24385.sort.rmdup.bam.

## Data features and quality assessment

Before discussing data applications, we first introduced metrics on quality assessment and then used the metrics to evaluate datasets sequenced for this study. Our focus will be on Hi-C, 10X, and haplotagging, which are currently or previously available on the market.

### Data metrics

In order to provide a robust analysis of the relative performance of the platforms, we first establish numerical metrics for evaluation. Since the Hi-C platforms differ significantly in the mode of operation from the Linked Read platforms, the metrics used will be slightly different, but we design them to enable as valid a comparison as possible.

#### Metric 1: association

Association refers to the ability of the platform to communicate with long-range information or, equivalently, the amount of non-local information contained within a read. Datasets with a higher association contain more and longer-range information than those with a lower association. In the context of using long-range information as an assembly tool, stronger association is preferable.

For the Hi-C platforms, association is measured by the distribution of link-separation distance, *i.e.*, the distance on the linear genome between the two ends of paired reads which have been linked together. If the first end of the pair aligns to a location i, and the second end to j, then the genomic distance is |i-j|. If large values of |i-j| are found to occur more often, then the dataset has a stronger association. Whilst we should therefore favour platforms which have a higher proportion of reads with large |i-j|, we note also that there is an expected pattern at higher distances: if the linkage probability is inversely proportional to the separation distances between read pairs with power b, then the long tail of the link-separation distance distribution is expected to fall approximately as:

(1) $$f\left(\left|i-j\right|\right)\propto {\left|i-j\right|}^{-b}$$

On a log–log scale, this manifests as a linear relationship between the separation distance and the observed frequency. Deviations from this pattern indicate problems with the library preparation and can result in the failure of any statistical inference based on the dataset. We should therefore prefer datasets which (1) exhibit a power-law relationship in frequency at high separation distances and (2) have a smaller exponent, resulting in a longer tail and hence more long-range information.

In the case of the Linked Read platforms, the long-range information is conveyed by labeling reads as originating from a larger molecule via a tag shared by all fragments of that molecule. The association should therefore be measured by the size of the molecules from which the labeled reads are drawn.

It is clear that having a larger molecule is generally better: each barcode delineates a larger spatial region, providing longer-range information. However, there is an upper limit beyond which increasing the molecule size gives decreasing returns. For example, if the molecules are at the chromosome scale, the barcoding would only indicates which chromosome the read originates from, which is useful but not beneficial for assembling the reads within a chromosome. Of critical concern, however, is the fact that increasing the molecule size would lead to an increase of the chances of barcode collisions, as demonstrated in Figure S2. Generally, the molecule size at which collision rates become untenable is significantly smaller than the genome size, and hence should be treated as the limiting factor. We should therefore favour platforms which generate longer molecule while maintaining a low collision rate.

#### Metric 2: accessibility

Accessibility refers to the fraction of the data which is unique, unambiguous, and useable. Datasets with low accessibility may still contain useful scientific data, but much more data would be required to achieve the same level of significance. We should therefore prefer platforms which produce highly accessible data. For example, both Hi-C and Linked Reads suffer from potential PCR duplication — the overamplification of some portions of the genome through the library preparation process. A high PCR duplication rate is indicative of a poor accessibility, and *vice versa*. Complex factors underlying the library preparation can also lead to reads which cannot be mapped to the reference genome (and the rate of unmapped reads is notably higher in long-range platforms than normal Illumina short reads), or which contain no linking information (“singletons”). Such “unmapped” reads contain no useful information, and thus should be excluded from further analysis.

In addition, Hi-C explicitly allows inter-chromosomal interactions (ICIs) to be mapped. Whilst this is useful generally in 3D genomics, for the purposes discussed in the “Applications” section, this represents unusable data, as assembly should occur on a per-chromosome basis. To maximize the amount of usable information, we should therefore prefer the platforms which have a smaller number of linkages between chromosomes, *i.e.*, a smaller ICI rate.

Assuming that other sources are negligible (or equal between platforms), the total accessibility of the dataset can therefore be computed from the PCR duplication rate D, the ICI rate C, and the unmapped rate U:

(2) A=1-D-C-U

A higher value of the total accessibility (A) indicates a dataset which contains more useful information.

#### Metric 3: evenness

Evenness is the measure of statistical validity in the coverage of the genome. A high coverage is evidently preferred, as it means that more portions of the genome are sampled and there is a smaller chance of missing portions of the genome; however, it is also important to ensure that the coverage is not biased onto some portions of the genome over others: there should be an equal likelihood of a read being generated anywhere on the genome. Datasets which deviate from this pattern are uneven, and likely biased in complex and unpredictable ways. We should instead seek out datasets with a higher level of evenness.

Under the standard statistical assumptions, if the genome is sampled at a uniform rate everywhere, the coverage should follow a Poisson distribution, $P\left(k\right|\lambda )$. However, it is easy to show that the coverage of any platform exhibits a significantly greater dispersion than a Poisson distribution with the correct mean. This is generally interpreted as being indicative that there is not a single rate, λ, at which the genome is sampled. Instead, there are multiple values, over which the distribution is marginalized [19].

In File S1, we use this information to generate the following unevenness metric:

(3) $$U=\frac{\text{Var}(\text{coverage})-〈\text{coverage}〉}{\text{Var}(\text{coverage})\;}$$

where Var(coverage) and $〈\text{coverage}〉$ are the standard statistical variance and the mean of the non-zero coverage distributions, respectively. This value is zero when the coverage distribution is a perfect Poisson distribution, and is arbitrarily large for distributions which have many values of λ contributing to them. We should therefore favour platforms which generate smaller values of U.

#### Metric 4: capability

Capability is the measure of usefulness of the dataset, *i.e.*, the ability for the dataset to improve the outcome of a genetic inquiry compared to what would be achieved without long-range information. A more capable platform, which produces data that significantly improve the assembly, should therefore be preferred.

We measure the capability by comparing the N50 and N90 metrics of a scaffolding with and without the assistance of long-range information. The N50 metric is the standard measure of “completeness”, and it is the length of the shortest continuous sequence such that all longer sequences make up more than 50% of the genome. N90 is defined similarly, but encapsulating 90%. Larger values for Nx are preferred, as this indicates that more of the genome has been grouped into larger, contiguous fragments.

### Hi-C reads

Of Hi-C technologies commercialized by three companies, *i.e.*, Cantata Bio (formerly Dovetail Genomics), Arima Genomics, and Phase Genomics, the most widely applied technologies are Omni-C (Cantata Bio) and Arima Genomics. In this study, we focused on analyzing Hi-C reads from Arima Genomics, and compared two generations of their technology (V1 and V2) to reveal the characteristics and library improvement of the platform. In total, we obtained three human datasets, two from V2 (NA24385-AJ and NA12878-CEU) and one from V1 (NA12878-CEU) (**Table 1**).

**Table 1:** Features of Hi-C reads from Arima Genomics in human datasets

Dataset	No. of read pairs	Unmapped rate (%)	PCR duplication rate (%)	ICI rate (%)	Total accessibility	N50 (Mb)	N20 (Mb)	N10 (Mb)
Arima V2 NA12878-CEU	352,429,304	20.9	6.8	12.7	0.596	47.9	96.3	130.3
Arima V2 NA24385-AJ	413,162,798	24.8	6.1	16.2	0.529	47.2	104.9	141.9
Arima V1 NA12878-CEU	415,173,112	28.6	10.1	18.5	0.328	28.2	63.1	100.0

*Note*: For each dataset, the total accessibility was calculated using Equation 2 based on three accessibility metrics — the unmapped rate, the PCR duplication rate, and the ICI rate. V1 and V2 indicate the two generations of the Arima Genomics Hi-C technology. Hi-C, high-throughput chromosome conformation capture; ICI, inter-chromosomal interaction; PCR, polymerase chain reaction.

**Figure 2:**
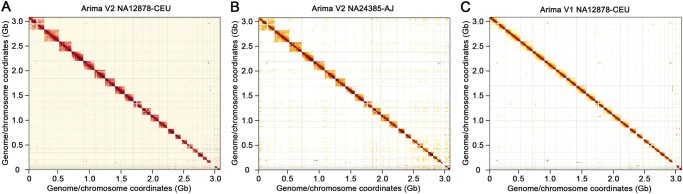
Hi-C maps for three human datasets **A**. Arima V2 NA12878-CEU. **B**. Arima V2 NA24385-AJ. **C**. Arima V1 NA12878-CEU. Each square block of the map represents an individual human chromosome, and darker region indicates higher contact density. These three datasets are all from female samples as there are hardly any contact interactions in chromosome Y.

**Figure 3:**
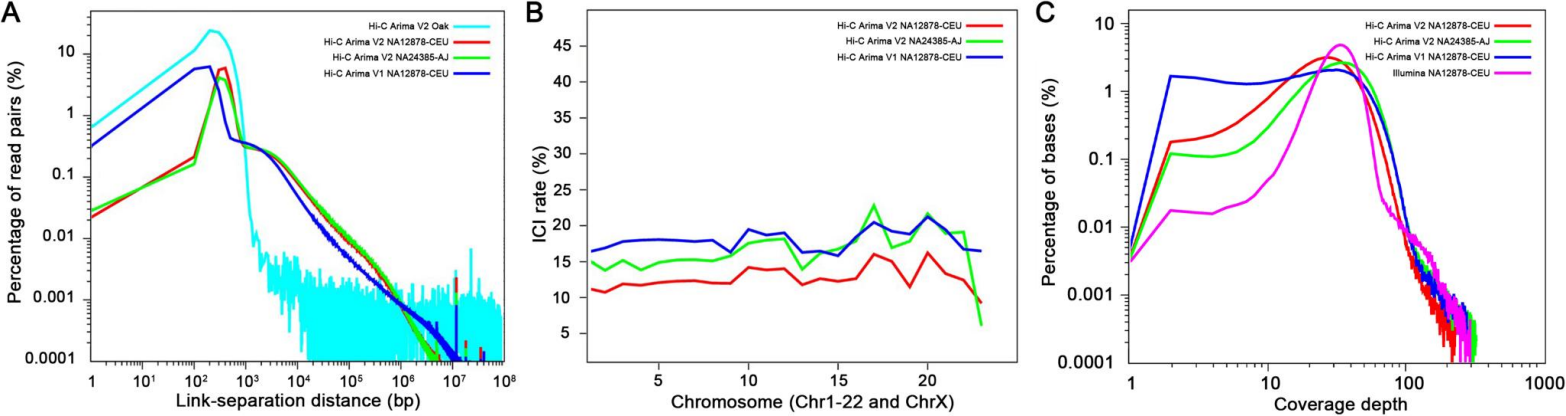
Characteristics of Hi-C reads **A**. Link-separation distance distribution: the distance in the linear genome between two reads which are coupled together by the Hi-C protocol, grouped into bins of 100 bp, and expressed as a percentage frequency. The Arima V2 Oak dataset is included, which demonstrates the breakdown of the power-law relationship of Equation 1, highlighting the desirable features present in the human datasets. The peaks which appear in all three human datasets at $\mathrm{LSD}\approx {1.085\times 10}^{7}$ are of unknown origins, though they are suspercted to be artefacts of the alignment methods used. **B**. ICI rate: the percentage of paired reads mapped to different chromsomes. The existance of inter-chromsomal pairs is not a desired feature for our purposes. In our quality control experiments, we set up a threshold of 30%, above which the dataset will be marked as failure. **C**. Base coverage distribution. Although all datasets are covered to approximately the same depth (30×), they show very different distributions around this value — a non-Hi-C dataset (Illumina) is included as a comparison. LSD, link-eparation distance; ICI, inter-chromosomal interaction.

Hi-C maps are shown in **Figure 2** for three human datasets by mapping the reads to the human reference assembly GRCh38. In these plots, regions of high density indicate real-space colocation of the genome, though there are some notable deviations. In particular, highly repetitive regions can cause spurious over-densities and under-densities, characterized by a cross-shape pattern, *i.e.*, the centromere of each chromosome. To explore the quality of these datasets in more detail, we presented **Figure 3** which consists of three separate plots showing link-separation distance (association), ICI rate (accessibility), and base coverage (evenness), respectively. Tabulated information regarding the metrics is also presented for accessibility (Table 1) and evenness (**Table 2**).

**Table 2:** Evenness statistics

Dataset		Coverage	Coverage variance	Unevenness
Human	Arima V2 NA12878-CEU	31.2×	195.0	5.3
	Arima V2 NA24385-AJ	38.8×	273.9	6.1
	Arima V1 NA12878-CEU	32.1×	363.1	10.3
	10X NA12878	57.2×	565.6	8.9
	Haplotagging	73.0×	446.0	5.1
	Illumina	35.1×	131.2	2.7
	stLFR NA24385	46.4×	250.8	4.4
Nonhuman	1X hummingbird	41.8×	98.6	1.4
	Haplotagging rat	83.3×	3715.1	43.6
	Haplotagging oak	90.64×	1133.9	11.5

*Note*: The unevenness metric was calculated using Equation 3 based on the mean and variance of the base coverage distributions shown in Figure 3C and Figure 5 (not downsampling). stLFR, single-tube long fragment read.

Figure 3A shows how the long-range information is distributed in the Hi-C datasets. As expected, we observed a peak of strongly associated regions around 100–500 bp (probably due to cross-linking failed read pairs, with the gap then being the insert size in the paired-end reads), and a long power-law tail for the three human datasets. For demonstration purposes, we also included an additional dataset derived from oak, which demonstrated a strong deviation from the power-law structure. In assessing the association demonstrated here, we would say that the oak dataset should be penalised due to this deviation, whilst the human datasets are comparatively much nicer. Additionally, the plots demonstrate that V2 datasets contain more information stored in longer reads than the V1 dataset, indicating stronger association.

The accessibility metric is shown graphically in Figure 3B and in more detail in Table 1. We found that the V1 dataset showed consistently poorer mapping rate, PCR duplication rate, and ICI rate than the V2 datasets, resulting in the total accessibility of 0.328, compared to 0.529 and 0.596 for the V2 datasets. However, as noted in Figure 3B, the difference between human datasets was, on some chromosomes, more pronounced than the difference between platforms.

Figure 3C shows the raw coverage data for the Hi-C data, along with a standard Illumina sequencing data of the NA12878-CEU sample for comparison. Given that the Illumina data has been sequenced more directly with fewer intervening biochemical alterations, we should expect it to be the “purest” sample. Visually, this appeared to be the case: the Illumina data were tightly peaked and resembled a Poisson distribution. The V2 datasets — though sampling to slightly different depths — showed a similar “fattened Poisson” distribution, and the V1 dataset seemed to be the least pure sampling, showing a strong over-density at low base coverage. These observations were carried through by the statistical metric developed in File S1, which are tabulated in Table 2: the Illumina dataset was given a score of 2.7, whilst the two V2 datasets scored 5.3 and 6.1, respectively, and the V1 dataset scored 10.3, indicating strongly uneven coverage. This indicates that whilst there is some statistical bias in the V2 datasets, it is significantly less than that of the V1 dataset.

Based on the information presented, we would conclude that the V2 platform produces data which robustly outperforms that from the V1 platform, with the two V2 datasets being very close together in quality. V2 NA24385-AJ has a slightly higher mean base coverage, but V2 NA12878-CEU scores slightly better on the total accessibility and evenness metrics. In the next section, we will demonstrate how Hi-C data can be used to aid genome scaffolding, and assess the capability of these datasets.

### Linked Reads

Based on data availability, we discussed the 10X, haplotagging, and stLFR qualities together, presenting an analysis on six datasets, including two from 10X (human and hummingbird), three from haplotagging (human, rat, and oak), and one from stLFR (human). The human 10X dataset was downloaded from the 10X Genomics website, hummingbird 10X dataset is part of the VGP project, and the human stLFR reads were downloaded from NCBI (see Data availability). The haplotagging datasets of human, rat, and oak were sequenced by the Sanger Institute as part of the DToL project. We note that, since the data arise from different species, we must take care with our inferences on the differences arising from the choice of platform rather than the choice of species. Any strong comparisons should be based primarily on the human samples.

**Figure 4:**
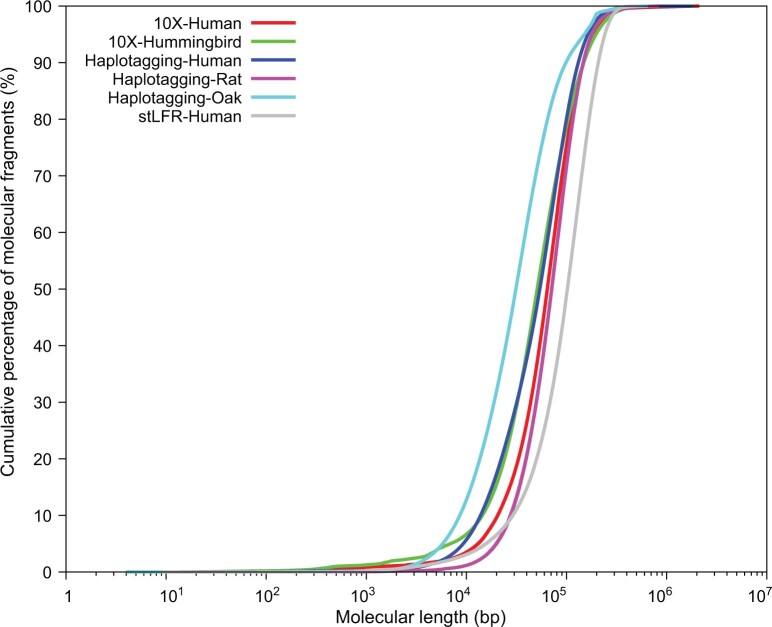
Length distributions for various 10X and haplotagging datasets Reads are grouped into fragments by barcodes, with shared barcodes identified and removed. The length of a fragment is the region covered by mapping coordinates from the Linked Reads which share the same barcode.

**Figure 4** shows the accumulated percentage of barcode-grouped reads by molecule length for the Linked Read plarforms. A leftward shift means longer barcode fragment for the same percentage of barcode-grouped reads. It is seems that stLFR has the best barcode length distribution, while haplotagging in oak is the worst, with other datasets falling in between. It should be noted that the read length for stLFR is 2 × 100 bp, while other datasets are in 2 × 150 bp. Table S1 shows the associated barcode collision frequencies for the molecule distributions. We find that all of the platforms produce molecules with collision rates below 0.1%, and have mean molecule lengths in the region of 50 kb. We note that the 10X platforms have a more prominent tail at the high-length end of the distribution, most evident through the N50 values: the 10X N50 values exceed the mean length by 40 kb, whilst the haplotagging N50 values exceed the mean length by only 20 kb. This indicates that the 10X includes a stronger tail of high-association data, even if the bulk of the data has similar associations.

**Table 3** shows the PCR duplication rates and the unmapped rates (and hence the accessibility) as well as N50 reads per barcode. It is clear that 10X has lower PCR duplication rates than haplotagging and stLFR, although the inverse is true for the unmapped rates. This is likely due to different tools used to analyze the datasets (EMA for haplotagging *versus* Long Ranger for 10X), and that the 10X is archival and several years old, whereas the haplotagging is state-of-the-art. Neverthelss, the total accessibility is broadly similar across the datasets. Here, the number of barcode N50 reads is an indicator of read density in single molecules, while the percentage of clustered reads is the portion of the reads assigned with unique barcodes. It is seen that 10X datasets show higher portions of clustered reads than those from haplotagging and stLFR datasets. The high PCR duplication rate for stLFR leads to a lower portion of the clustered reads.

**Table 3:** Features of 10X, haplotagging, and stLFR datasets

Dataset	Platform	Tool	No. of read pairs	Unmapped rate (%)	PCR duplication rate (%)	Total accessibility	Molecular length (N50, Mb)	No. of barcode N50 (≥ 5) reads	No. of barcode N50 (≥ 3) reads	Percentage of clustered reads (%)
Human NA12878	10X	LR+S	669,583,370	10.1	21	0.689	94,611	81	80	88.2
Hummingbird	10X	LR+S	159,605,373	14.9	5	0.801	63,292	22	21	87
Human NA12878	Haplotagging	EMA+S	678,683,208	2.52	30.7	0.678	73,294	12	11	55
Rat	Haplotagging	EMA+S	742,824,305	2.5	30	0.675	78,250	11	11	50
Oak	Haplotagging	EMA+S	208,869,403	4.17	39.5	0.563	54,969	11	8	50
Human NA24385	stLFR	Unknown	2,525,286,352	0.002	40.2	0.598	102,454	128	128	59.8

*Note*: Long Ranger and EMA are alignment tools for Linked Reads, details see https://github.com/10XGenomics/longranger and https://github.com/arshajii/ema. LR, Long Ranger; S, SAMtools.

**Figure 5:**
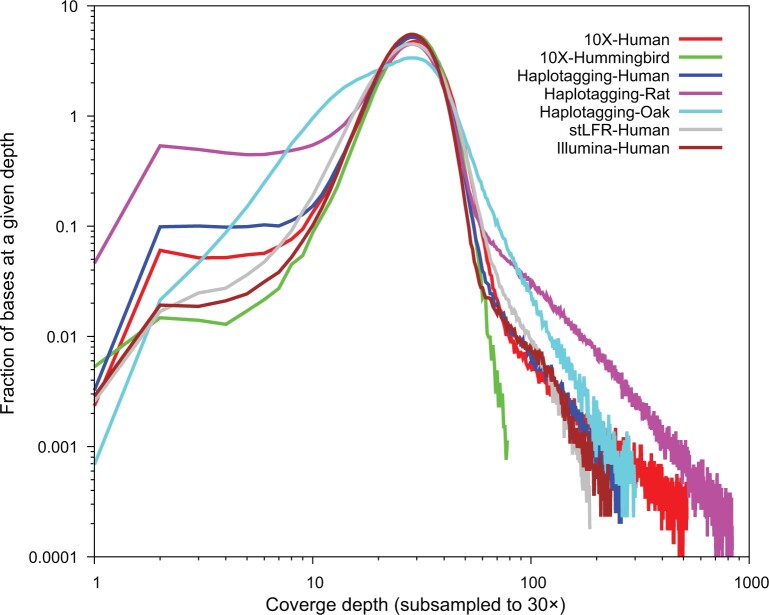
Base coverage profiles for various 10X and haplotagging datasets The 10X and haplotagging downsampled datasets at ∼ 30× are used to remove the effects of differing coverage depths. In terms of coverage evenness, the datasets of rat and oak are not as smooth as other samples.

**Figure 5** shows the coverage profiles of the Linked Read datasets in a 30× downsampled form to eliminate the effects of differing sequencing depths. The coverage is given as a fraction of the maximal value. In Figure 5, we found a number of clear features. It is clear that both the oak and rat profiles show extremely strong deviations from an even profile. This may be due to the effects such as highly repetitive regions (which rats are known to possess [20]), which cause some regions of the genome to be erroneously “covered” thousands of times while other regions are deprived of coverage. The human profiles are similar in shape to the Illumina curve, while their high-coverage ends (as with the rat profile, likely a spurious tail due to over-coverage of repetitive regions) are suppressed relative to the Illumina sample, indicative of the long-range information allowing correct alignment of some repetitive regions. However, we observed a stronger bias toward the low-coverage ends in both the 10X and haplotagging data than those in Illumina and stLFR data. In contrast, the hummingbird profile displays a remarkably Poisson-like shape, largely due to the almost total absence of a repetitive high-coverage tail — likely a feature of a small, non-repetitive genome [21].

These visual conclusions are supported by the unevenness metrics shown in Table 2: both the rat and oak datasets score very poorly (43.6 and 11.5, respectively), the human datasets score between 2 and 9 (Illumina: 2.7, haplotagging: 5.1, and 10X: 8.9), and the hummingbird dataset has the lowest score of 1.4. We note that the haplotagging’s improved score over the 10X platform likely reflects its more effective suppression of the over-coverage of repetitive regions, rather than of an improvement at the low-coverage end. This might indicate that haplotagging is more successful in aligning repetitive regions than 10X.

From the metrics presented here, we conclude that, for base polishing, 10X data are superior to haplotagging data due to their slightly higher association strength. However, haplotagging has a larger number of unique barcodes, resulting in a much lower collision rate, making it more efficient to handle large number of samples when targeting low-coverage data. In addition, the statistical properties of the haplotagging platform indicate that haplotagging allows better alignment of highly repetitive regions than 10X. For stLFR, the read length of 2 × 100 bp is notablly shorter than that of 2 × 150 bp of 10X and haplotagging. If the same read length is sequenced and PCR duplication rate can be reduced, it is possible that stLFR could be the best platform.

### TELL-seq

TELL-seq could be a promising successor to 10X technology, and we performed a similar analysis of the platform using the metrics we have formulated. However, the authors were unable to produce libraries of sufficient quality to provide a viable comparison.

When assessing the use in various applications, we must rely on existing literature [11,18,22], rather than developing our own independent quality metrics. It is noted that the TELL-seq platform claims comparable — and in some cases superior — performance to the 10X platform. As the technologies continue to be developed, we hope that the metrics presented here will be applied to a wide range of platforms, providing a robust basis for comparisons.

## Applications

In this section, we briefly outlined some of the main applications for long-range data, and discussed how these have been applied in the literature.

### Genome scaffolding

Genome scaffolding is the process by which a number of continuous sequences (“contigs”) generated from overlapping reads are linked together into a single structure (a scaffold) of known sequences. These sequences are separated by gaps of unknown sequences, but the length of the gap is relatively well constrained. This forms a critical step in genome assembly [22], but conventional means are both laborious and computationally intensive. Recent advances in long-range sequencing technologies have improved the continuity of genome scaffolds [23]. For example, the assembly quality thresholds proposed by VGP are that contig N50 is > 10 Mb and the scaffold N50 is the chromosome length [24], indicating that chromosome-scale scaffolding is now routinely achievable.

#### Genome scaffolding with Hi-C reads

The Hi-C protocol provides a fast and lower-cost way of constructing scaffolds from the contigs. The spatial information within Hi-C reads can identify whether contigs come from the same chromosome and infer the correct orders of the contigs within each chromosome based on the relative proximity between bases in each contig [13]. This technology is widely used to assemble the contigs of eukaryotic genomes into chromosome-scale scaffolds [22,24], and has recently been applied to achieve a chromosome-level assembly of the giant and complex genome of Chinese pine [25]. To further improve the quality of genomic assembly, some studies evaluated different sample preparation kits/protocols and computational programs, and identified the optimal conditions for Hi-C scaffolding [26].

**Figure 6:**
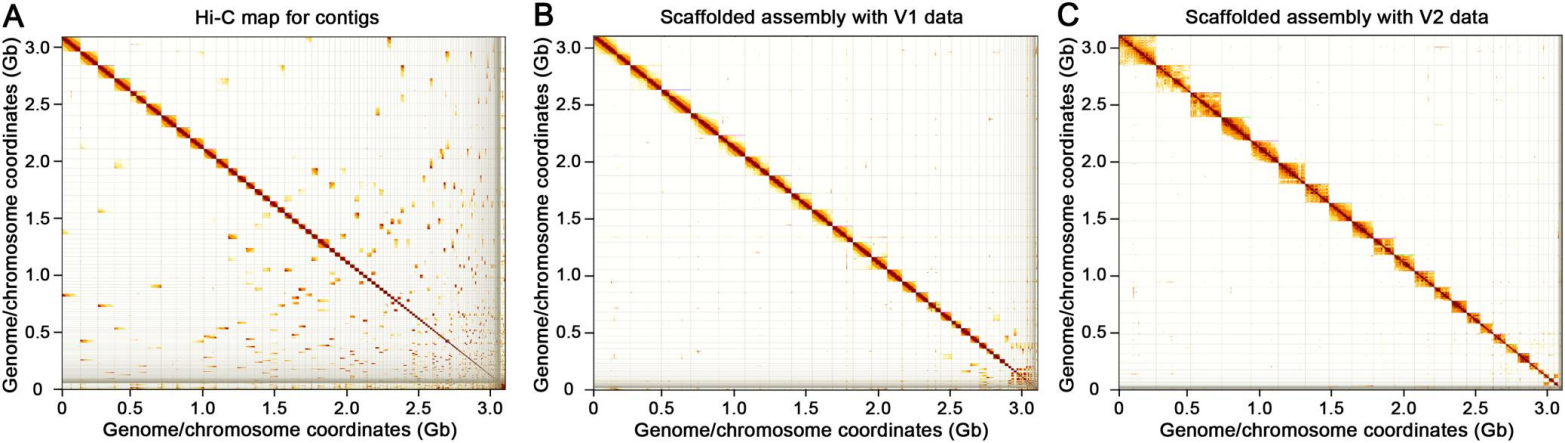
Hi-C maps on contigs and scaffolded assemblies **A**. Contig fragmentation is clealy observed in the Hi-C map. **B**. Assembly with Arima V1 data. A chromosome-level assembly is in shape, while some small contigs can still be obsevrved in the lower-right corner. **C**. Assembly with Arima V2 data. A much improved chromosome-level assembly is observed.

To demonstrate how Hi-C data can improve the quality of scaffolding, we applied the abovementioned methods to the human genome. 54× PacBio HiFi reads from HG002 were downloaded from Genome in a Bottle (GIAB) and an assembly was obtained using Hifiasm [27] with contig N50 at 45.1 Mb. This represents the baseline shown in **Figure 6**A, which is a standard Hi-C proximity map. However, without scaffolding, the proximity map shows a high degree of fragmentation. After removing haplotype duplications, the contigs were further assembled to chromosome-level scaffolds using ∼ 30× Hi-C reads and the YaHS scaffolding tool [28]. The resulting maps for Arima Genomics V1 (Figure 6B) and V2 (Figure 6C) are shown. After scaffolding, chromosome blocks are clearly seen and the fragmentation is visibly reduced. **Table 4** details how the lengths of the assembled fragments vary after applying the Hi-C data. Although the V1 platform produces slightly higher N50 scaffolds and a larger maximum length scaffold, the V2 platform has a higher mean length, indicating a significant reduction in the number of poorly-scaffolded contigs, consistent with our earlier analysis of these platforms.Instructions in details on genome assemblies are provided in File S2. Assembly pipelines/instructions/recommendations can also be found in VGP [24].

**Table 4:** Assembly statistics

Data and assembly	Total length (Gb)	No. of sequences	Sequence length (Mb)			
			Mean	N50	N90	Maximum
HiFi reads of HG002	167.76	14,949,433	0.011	0.011	0.009	0.021
Contigs with Hifiasm	2.866	1126	2.54	45.1	6.93	116
Contigs after Purge_Dups	2.83	434	6.52	45.1	8.46	116
Scaffolds with Arima V1	2.83	162	17.5	152	78.4	324
Scaffolds with Arima V2	2.83	152	18.6	144	75.12	235

*Note*: The table shows the statistics from contigs to scaffolds. After Purge_Dups, a small portion of the assembly has been removed. Comparing with Arima V2, there is a notable mis-assembly error in Arima V1 as its maximum length is longer than the chromosome length.

Along with Hi-C genome scaffolding, Strand-seq is another datatype which can produce chromosome-scale, haplotype-resolved assembly of human genomes. Strand-seq is a single-cell sequencing method which preserves structural contiguity of individual homologs in every single cell [29]. When contigs from long reads are available, Strand-seq data can be used to order and orient contigs into chromosomes. Good results have been reported as the datatype provides sequence strand information. However, this method requires active cell culture and single-cell isolation, which limit its applications to a wide range of species for *de novo* assemblies. As a result, the majority samples in VGP and DToL do not have Strand-seq data for scaffolding.

#### Genome scaffolding with Linked Reads

The Linked Read strategy provides an effective and less expensive alternative technology than NGS mate-pair data for genome scaffolding [22], since the nature of these platforms groups reads by their proximity in the genome (absent any barcode collisions). The information retained from long stretch sequences can be used to link different contigs to chromosome-level scaffolds, a strategy which is already widely used for genome scaffolding in a variety of complex and polyploid species. For example, an early study applied 10X reads to assist in scaffolding of genome sequence of *Triticum urartu*, the progenitor of the A subgenome of tetraploid and hexaploid wheat [30]. Additional, Lee et al. [31] reported that 10X Linked Reads were successfully used to correct and scaffold the assembly for an allopolyploid rapeseed.

Currently, 10X-based approaches can no longer be used due to the withdrawal of the product, but alternative Linked Read technologies have developed based on similar methods, such as haplotagging, stLFR, and TELL-seq [11,12]. As we have seen, the association strength between these platforms is similar, and hence these platforms all offer the ability to produce high quality scaffolding. This assumption was validated when Xu et al. [32] used stLFR for scaffolding in the assembly of porcupinefish, and Chen et al. [11] completed a comprehensive assessment of TELL-seq using the sequencing data from samples NA12878 and NA24385. The TELL-seq data of NA12878 produced a *de novo* assembly with a scaffold N50 of 31.5 Mb, the longest contig of 109.2 Mb, and the longest alignment of 23.6 Mb. The stLFR platform is a well-established Linked Read technology that can leverage DNA co-barcoding information to connect contigs and improve the precision of genome assembly. Furthermore, stLFR has also been employed for polishing and gap filling in genome assemblies [33,34].

### *De novo* genome assembly

*De novo* genome assembly is the fundamental process in reconstructing a genome from sequencing reads without a reference sequence [35]. A whole-genome assembly with high level of completeness, continuity, and accuracy is crucial, which can significantly enhance the reliability of the downstream analyses. In general, the primary step for *de novo* assembly from the collection reads consists of three phases, contig assembly, scaffolding, and gap filling. As previously noted, both Hi-C and Linked Reads can connect and order contigs into a “scaffold” in the second phase. However, Linked Reads also offer additional support for the other procedures involved in *de novo* assembly, due to their inherently more structured nature.

10X technology has been used extensively for the *de novo* assembly of the eukaryotic and prokaryotic genomes [3]. For instance, the first complete genome sequence for the mound-building mouse, *Mus spicilegus*, was generated with 10X reads and resulted in the *de novo* assembly of a 2.50-Gb genome with a scaffold N50 of 2.27 Mb [36]. Using 10X data and the Supernova assembler, Ozerov et al. [37] assembled a ∼ 0.8-Gb draft genome of *Silurus glanis*, an important species for freshwater ecosystem balance. It has also been demonstrated that 10X data can be used to assemble high-accuracy contigs and scaffolds, even for large polypoid plant genomes with highly similar repetitive sequences [3,38].

As comparatively newer technologies, other Linked Read platforms have seen less ubiquitous use in *de novo* assembly, though Chen et al. [11] introduced the TELL-seq platform by immediately providing *de novo* assembly of bacteria (*Escherichia coli*, *Campylobacter jejuni*, and *Rhodobacter sphaeroides*) and humans (NA12878). The human assembly showed “longer aligned contig length and at least 28% and 71% fewer misassemblies than other Linked Read or nanopore methods, respectively” [11]. Gao et al. [39] utilized stLFR and Hi-C sequencing technologies to achieve *de novo* chromosomal-level assembly of the rough-toothed dolphin. A recent effort in using stLFR for contig scaffolding and genome assembly has been reported [40].

Although *de novo* genome assembly can be performed by using Linked Read technologies alone, most current studies adopt a hybrid strategy of multiple technologies to complete genome assembly. For example, Batra et al. [41] performed a *de novo* genome assembly of the olive baboon by using a hybrid sequencing approach of 10X sequencing, Oxford nanopore sequencing, Illumina paired-end sequencing, and Hi-C, which have complementary advantages. Lind et al. [42] generated a high-resolution *de novo* chromosome-scale genome assembly for the Komodo dragon *Varanus komodoensis* using data from different platforms, including 10X, Oxford nanopore long reads, PacBio long reads, and BioNano optical mapping. Zhang et al. [43] used a hybrid strategy of stLFR and Oxford Nanopore Technologies (ONT) technologies to assemble the first high-quality genome of Ossabaw pig with a contig N50 of ∼ 6.03 Mb, which is significantly higher than most other published pig genomes.

### Variation detection

One of the most fundamental goals in genetics is to link genomic variations and the evolution of traits between populations or species. DNA polymorphisms are widespread genomic variations among individuals and include single-nucleotide variations (SNVs), small insertions and deletions (Indels; < 50 bp), and structural variations (SVs). Many methods have been proposed to test DNA changes across the genome from different sequencing technologies, but there are still considerable limitations on what can be achieved in SV detection due to technical difficulties of the standard short-read platform. The long-range information provided by Linked Read and Hi-C platforms can improve detection for haplotype-specific deletions and large SVs [44,45].

#### Variation detection with Hi-C reads

Since Hi-C technology detects regions of high interaction probability in a genome, this intrinsically makes it particularly useful for detecting SVs. One of the main advantages of Hi-C is that it can accurately detect SVs with low-depth sequencing data. This feature provides a higher chance of identifying SVs at repetitive regions in complex genomes.

As a result, Hi-C has been demonstrated to be a promising technology to precisely detect SVs, including chromosomal rearrangements and copy number variation (CNVs) in plant and human genomes [46,47]. In recent years, several research projects have shown the ability of Hi-C to support identifying 3D genome organization alterations as a result of SVs in the human cancer genome [48–50]. Hi-C has also been applied to screen the complex genomic rearrangements associated with the development of disease in humans. For example, Hi-C was used to investigate the genetic variation that causes developmental disorders [51], and detect multi-megabase polymorphic inversions in wheat and barley [52,53].

#### Variation detection with Linked Reads

Recent work has used SNVs detected by 10X sequencing technology to draw the landscape of meiotic recombination in plant populations at the genome-scale resolution [54,55]. Rommel Fuentes et al. [55] pinpointed meiotic crossovers of interspecific hybrid F1 tomato pollen at the SNV resolution by using 10X data. This technique also has been a powerful tool for detecting genomic variants associated with human diseases. A number of novel and important SVs associated with metastatic castration-resistant prostate cancer were identified by 10X whole-genome sequencing [56], and Linked Read sequencing validated the inverted rearrangement in the triple-negative breast cancer sample TN-19 [57]. A study in 2020 confirmed that 10X sequencing provides a cost-efficiency way of mining genomic variants at moderate depth and population scale [58]. It was also reported that 10X technology could be used to screen nucleotide resolution of the SVs linked with potential risk loci in small and rare disease cohorts [59].

Haplotagging is particularly suitable for constructing the original haplotype and has been successfully applied to construct the genome haplotypes in the two butterfly species, identifying the genetic markers controlling the distinct wing color patterns [12]. This indicates that haplotagging might be a promising method to identify the superior haplotype alleles in the diverse plant or animal populations for model and non-model species. Bhat et al. [17] thought that this technique would provide important support for haplotype-based breeding for crop improvement.

The utility of the TELL-seq technology for detecting genome variations has not been nearly so widely used in plants or animals, though the study by Chen et al. [11] demonstrated that Linked Reads generated by TELL-seq could be used to screen genetic variations using an analysis pipeline developed for the 10X technology. Although this means that TELL-seq could also be used to detect SVs, the initial study found that it failed to identify some deletions in the NA12878 sample. The authors thought that two factors (the short library insert length and different barcoding chemistry) might be responsible, and they encouraged the research community to further develop and optimize analytical tools to improve the ability to detect SVs when using Linked Reads [11].

Similarly, the initial study on stLFR indicated that its use could enhance the accuracy of detecting genomic variants with further optimization [18]. It was also observed that stLFR outperformed in SV detection and successfully identified known translocations in human samples as well as the types of SVs.

More extensive validation studies are therefore needed to prove whether stLFR and TELL-seq can be alternate methods for the 10X platform in accurately detecting genome-wide variations. Such efforts will no doubt be facilitated by the specialised software tools which continue to be released for these platforms.

### Other applications

#### Phasing

Phasing, the assignment of alleles to either the maternal or paternal haplotype, is another potential application for long-range reads, since even long reads can struggle to accurately identify heterozygosity and correctly assign differences to haplotypes. Along with *de novo* assembly, Chen et al. [11] demonstrated how TELL-seq can be used as a powerful tool for phasing the genome. Their TELL-seq phasing results on the NA12878 and NA24385 samples showed that the highest heterozygous rates are 99.9% and 99.8%, the phasing block N50 sizes are 16.1 Mb and 13.4 Mb, the longest phasing blocks are 67.5 Mb and 59.2 Mb, and the lowest switch error rates are 0.04% and 0.08%, respectively [11].

Most recently, a study compared the performance and accuracy of genome phasing between Hi-C and 10X in Hanwoo cattle [60]. This study found that the phasing strategy with 10X technology and Long Ranger software displayed the best phasing performance. The best strategy had the highest phasing rate (89.6%), longest adjusted N50 (1.24 Mb), and lowest switch error rate (0.07%). Moreover, the phasing accuracy and yield of the best strategy stayed over 90% for distances up to 4 Mb and 550 kb, respectively.

The stLFR technology has also been successfully used in alignment-based haplotyping. According to the study by Wang et al. [18], stLFR demonstrated excellent phasing performance: over 99% of all heterozygous SNPs were accurately phased and placed into phase blocks with N50 ranging from 1.2 Mb to 34.0 Mb in the human genome of the NA12878 sample. However, the performance of stLFR in genomic phasing depends on the library type and the coverage of sequence data. Xu et al. [32] applied HAST tool to stLFR sequencing data generated from an Asian individual, resulting in a assembly with high haplotyping precision (∼ 99.7%) and recall (∼ 95.9%) covering 94.7% of the reference genome with a scaffold N50 longer than 11 Mb.

#### Metagenomics

Another application of Linked Read sequencing technologies is assembling high-quality metagenome of microbial species, which is able to improve continuity and accuracy in *de novo* assembly using barcode information, as comprehensively evaluated by Zhang and his colleagues [61]. This study showed that 10X reads significantly improved the metagenome assemblies when compared with Illumina short reads, although PacBio HiFi long-reads outperformed both platforms. Due to the low cost and the high base quality, sequencing metagenomes using Linked Read technologies remains persuasive. Recently, Roodgar et al. [62] explored the longitudinal trajectories of gut microbiome for a single individual using Linked Read metagenomic sequencing in 10X Genomics Chromium platform. Qi et al. [63] proposed a strategy called MetaTrass that utilizes stLFR technology and public reference genomes to recover high-quality, species-resolved human gut microbiomes.

## Software tools

To date, a large number of tools have been developed to analyze data generated from long-range sequencing technologies [61,64,65]. Here, we highlighted recent developments in software tools used for genome scaffolding, *de novo* assembly, and variation detection based on the long-range linking information.

### Hi-C analysis tools

#### Tools for genome scaffolding with Hi-C reads

Several scaffolding methods have been developed for assembling contigs into scaffolds based on Hi-C data, examples of which are shown in **Table 5**. Many different approaches can be taken in designing these tools, which we broadly categorize into three groups: deterministic, probabilistic, and improver.

**Table 5:** Analysis tools for long-range reads

Application		Software tool	Year	Property	URL	Refs.
Hi-C	Genome scaffolding	LACHESIS	2013	Deterministic, agglomerative hierarchical clustering	https://github.com/shendurelab/LACHESIS	[66]
		dnaTri	2013	Deterministic, agglomerative hierarchical clustering	https://github.com/NoamKaplan/dna-triangulation	[67]
		GRAAL	2014	Probabilistic MCMC	https://github.com/koszullab/GRAAL	[71]
		instaGRAAL	2020	Probabilistic MCMC, refined for large genomes	https://github.com/koszullab/instaGRAAL	[72]
		SALSA2	2019	Novel iterative scaffolding method	https://github.com/marbl/SALSA	[64]
		3D-DNA	2017	Deterministic best-neighbor, mega-scaffold approach	https://github.com/aidenlab/3d-dna	[69]
		HiRise	2016	Maximum likelihood algorithm, official Dovetail product	https://github.com/DovetailGenomics/HiRise_July2015_GR	[73]
		ALLHiC	2019	Deterministic hierarchical clustering on autopolyploid or heterozygous genomes	https://github.com/tangerzhang/ALLHiC	[68]
		HiC-Hiker	2020	Probabilistic, dynamic programming approach to improve quality of already-scaffolded data	https://github.com/ryought/hic_hiker	[74]
		EndHiC	2021	Improve quality of already-scaffolded data	https://github.com/fanagislab/EndHiC	[75]
		YaHS	2022	Probabilistic, novel inference algorithm	https://github.com/c-zhou/yahs	[28]
	Variation detection	HiCnv	2018	Detect CNVs using HMMs	https://github.com/ay-lab/HiCnv	[48]
		OneD	2018	Detect CNVs using HMMs	https://github.com/qenvio/dryhic	[77]
		HiCtrans	2018	Detect translocations using HMMs	https://github.com/ay-lab/HiCtrans	[48,78]
		HiNT	2020	Detect both CNVs and translocations	https://github.com/parklab/HiNT	[78]
		HiTea	2021	Identify mobile transposable element insertions	https://github.com/parklab/HiTea	[79]
		NeoLoopFinder	2021	Find SV-induced chromatin loops	https://github.com/XiaoTaoWang/NeoLoopFinder	[80]
		EagleC	2022	Deep-learning method for full-spectrum SV detection	https://github.com/XiaoTaoWang/EagleC	[47]
Linked Reads	Genome scaffolding	fragScaf	2014	Assembly via transposase contiguity	https://github.com/adeylab/fragScaff	[81]
		Architect	2016	Agglomerative hierarchical clustering	https://github.com/kuleshov/architect	[86]
		ARCS	2018	Designed specifically for 10X	https://github.com/bcgsc/arcs	[82]
		ARKS	2018	*K*-mer mapping for improved efficiency in ARCS	https://github.com/bcgsc/arks	[83]
		ARBitR	2021	Explicitly designed for multiple Linked Read platforms	https://github.com/markhilt/ARBitR	[84]
		SLR-superscaffolder	2021	Divisive hierarchical clustering	https://github.com/BGI-Qingdao/SLR-superscaffolder	[85]
	*De novo* assembly	Supernova	2017	Official 10X assembly product	https://support.10xgenomics.com/de-novo-assembly/software/overview/latest/welcome	[15]
		cloudSPAdes	2019	De Bruijin assembler, extensible to metagenomic or hybrid data	https://github.com/ablab/spades/releases/tag/cloudspades-paper	[89]
		Ariadne	2021	cloudSPAdes module, deconvolve barcodes accurately	https://github.com/lauren-mak/ariadne	[90]
		Athena	2018	Improve metagenomic assembly	https://github.com/abishara/athena_meta	[91]
		TuringAssembler	2020	Introduced explicitly for TELL-seq data	https://github.com/bioturing/TuringAssembler	[11]
	Variation detection	Long Ranger	2019	Official 10X variation detection tool, use augmented GATK approach	https://support.10xgenomics.com/genome-exome/software/downloads/latest	[45]
		GROC-SVs	2017	Similar to Long Ranger, use local assembly to improve resolution	https://github.com/grocsvs/grocsvs	[44]
		Aquila	2021	Identify genetic variations in personal genomes	https://github.com/maiziex/Aquila	[97]
		Aquila_stLFR	2021	Designed specifically for stLFR data	https://github.com/maiziex/Aquila_stLFR	[98]
		AquilaSV	2021	Detect SVs from Linked Read data	https://github.com/maiziezhoulab/AquilaSV	[22]
		NAIBR	2018	Probabilistic model using “split molecule” approach	https://github.com/raphael-group/NAIBR	[99]
		VALOR	2017	Detect genomic inversion from “split molecule” signature	https://github.com/BilkentCompGen/valor	[100]
		VALOR2	2020	Expanded from VALOR to detect more types of SVs	https://github.com/BilkentCompGen/valor	[101]
		ZoomX	2018	Novel probabilistic approach to detect large rearrangements	https://bitbucket.org/charade/zoomx/src	[103]
		LEVIATHAN	2021	Detect SVs in highly fragmented and heterozygous genomes	https://github.com/morispi/LEVIATHAN	[96]

*Note*: MCMC, Markov Chain Monte Carlo; CNV, copy number variation; HMM, hidden Markov model; SV, structural variation; URL, uniform resource locator; SLR, synthetic long read.

Deterministic tools use algorithms which always return the single result which optimizes some underlying metrics. Some examples of deterministic algorithms are as follow. (1) Hierarchical clustering. It typically uses an agglomerative approach, such as those in the early tools LACHESIS [66] and dnaTri [67] (both no longer actively developed) and a more recent tool ALLHiC (a framework particularly designed for scaffolding autopolyploid or heterozygous diploid genomes) [68]. (2) Best-neighbor. Though deterministic, best-neighbor returns only an approximation of the true optimum, offering the benefit of vastly increased speed. For example, 3D-DNA [69] used this approach after correcting the input contigs to assemble the contigs into one mega-scaffold before it is then cuts into a number of chromosomes based on the Hi-C contact matrix. (3) Maximal matching. This approach is used in the SALSA1 [70] tool, which first corrects misassemblies derived from the input contigs using a low Hi-C mapping rate as an error signal and then orients and orders the corrected contigs to generate scaffolds using a maximal matching algorithm. (4) Novel approaches. More novel solutions include SALSA2 [64], an overhaul of SALSA1 that utilizes all the interaction information from the Hi-C map to reduce assembly errors through a novel iterative scaffolding method, and the newly developed YaHS [28], which introduces a novel algorithm to establishing the contact matrix to obtain more accurate inferences of contig joins.

Probabilistic approaches, in contrast, return results which are not exact, but provide good approximations to the desired solution when direct computation would be prohibitive. We identified two main classes of probabilistic algorithms. (1) Markov Chain Monte Carlo (MCMC). The methods are a class of algorithms which attempt to efficiently approximate drawing values from an underlying (unknown) distribution function. This algorithm is used by GRAAL [71], which employs an MCMC algorithm to generate scaffolds from the Hi-C data. Recently, Baudry et al. [72] developed instaGRAAL, an upgrade version of GRAAL, capable of assembling large genomes. (2) Maximum likelihood. The maximum likelihood methods use Bayesian formulations to derive a probability of observing a given result based on a hypothesized original state. By optimizing this function, the original state can be inferred. This approach is used by HiRise, the tool developed by Dovetail Genomics for their Hi-C service [73].

Finally, we noted a class of tools referred to as improvers, since these tools do not perform the assembly themselves but enhance the quality of assemblies generated by other tools. Examples include HiC-Hiker [74], a probabilistic and dynamic programming approach which can improve the quality of scaffolds produced by other Hi-C scaffolding software, and the recently developed EndHiC [75], which can reduce the error rate of assembly using only the the Hi-C contacts from the end regions of the contigs.

Several studies have evaluated the performance of different scaffolders for scaffolding accuracy [28,68,75,76]. For example, a recent study evaluated the performance of five Hi-C scaffolders including LACHESIS, HiRise, 3D-DNA, SALSA2, and ALLHiC. The results showed that the HiRise and LACHESIS displayed the best performance on average across all tested scripts [76]. However, for all the available software, it remains challenging to correctly assemble large contigs into chromosomes, and manual checking and curation are often necessary. The selection of suitable tools therefore often remains an exercise in trial-and-error by the researcher.

#### Tools for variation detection with Hi-C reads

Several computational tools have been developed to identify SVs from chromatin interaction data. We divided these tools based on the types of SVs they can detect.

Tools which can identify CNVs include HiCnv [48], OneD [77], and HiNT-CNV [78]. Generally speaking, these tools use Bayesian information criteria [and in the case of HiCnv and OneD, hidden Markov models (HMMs)] to identify the location of CNVs. Similar methods can be used to identify inter-chromosomal translocations — the tools HiCtrans and HiNT-TL are packaged alongside HiCnv and HiNT-CNV, respectively [48,78]. Although the abovementioned algorithms were used to screen the SVs within Hi-C data, most of these methods can only detect inter-chromosomal translocations and long-range intra-chromosomal SVs at a low resolution.

More specific tools include HiTea [79], developed specifically for identifying mobile transposable element insertions in Hi-C data, and NeoLoopFinder [80], developed for predicting SV-induced chromatin loops and also capable of detecting complex SVs with Hi-C data. Wang and colleagues [47] have also presented a computational framework, EagleC, which integrates deep-learning and ensemble-learning strategies to detect a full range of SVs at a high resolution.

Overall, there are still strong demands for analysis tools that can use Hi-C data for high-resolution SV detection.

### Linked Read analysis tools

#### Tools for genome scaffolding with Linked Reads

Generally speaking, most Linked Read tools should be equally effective, regardless of which platform was used. However, due to its prominence, many tools were designed specifically for 10X, and so their applicability to other Linked Read platforms, including TELL-seq and haplotagging, still needs to be further verified. Unlike the Hi-C tools which use a wide variety of different algorithms for scaffolding, Linked Read algorithms broadly follow the same approach: first attempt to unambiguously identify the HMW-DNA fragments each read originated from, and then use these fragments as the basis for a scaffolding.

fragScaff [81] was originally developed for scaffolding the data from contiguity preserving transposase sequencing, but was one of the first tools to receive explicit support for 10X reads. fragScaff uses an explicit threshold metric to determine barcode uniqueness, before constructing and traversing a scaffold graph. ARCS [82] and ARKS [83] are two closely related tools developed by the same team: ARCS is a stand-alone genome scaffolding tool developed specifically for 10X, whilst ARKS uses a *k*-mer mapping strategy to align Linked Reads and contigs to improve computational efficiency, and is an optional additional mode for ARCS. Hiltunen et al. [84] presented a software package ARBitR, which is explicitly designed to work on multiple platforms beyond 10X. The main distinctive feature of the ARBitR is that it considers the overlaps between the involved contigs when splicing, so as to improve the genome scaffolding accuracy.

Other Linked Read tools include SLR-superscaffolder [85], which uses an inverted top-down approach, and Architect [86], which uses co-barcoding and paired-end information to improve the contiguity of genome scaffolding.

#### Tools for de novo assembly with Linked Reads

Although there is much mature software that can be applied to *de novo* assembly of genomes with short-read sequencing data [35,87,88], comparatively fewer tools have been developed for generating a *de novo* genome from Linked Read data.

Supernova [15], developed by 10X Genomics, is specifically designed for *de novo* assembly of genomes using deeply sequenced data from 10X sequencing platform. Compared with other methods, Supernova can generate phased diploid assemblies over very long distances. Moreover, despite being a 10X product, Supernova can also be used for the data generated on other platforms, such as TELL-seq [11].

Other assembly tools often use a de Bruijin graph-based approach. For example, cloudSPAdes [89] (an extensible module of the SPAdes assembler) uses Linked Read data to expand the de Bruijn graph, and can also be applied to metagenomic or hybrid assembly. The Ariadne [90] module uses a novel algorithm based on de Bruijin graphs to handle the barcode deconvolution problem. In their introduction of the TELL-seq platform, Chen et al. [11] presented TuringAssembler, another de Bruijn graph-based assembler.

Whilst not strictly related to *de novo* assembly, we also noted that Bishara et al. [91] presented an assembler, Athena, which uses the tag information from Linked Read sequencing to improve metagenome assembly.

#### Tools for variation detection with Linked Reads

A number of tools developed to detect genetic variations in NGS data can also be used on Linked Read data without significant modification, such as GATK [92], SNVer [93], VarScan [94], and VarDict [95]. However, since these tools do not exploit the long-range information, their genome-scale SV detection remains limited, and tools which are aware of the long-range information promise much greater detection power.

Long Ranger [45] is the official program developed by 10X Genomics, which can screen variants and SVs, and combines a number of existing tools, such as BWA and GATK, augmented with long-range specific algorithms. GROC-SVs [44] adopts a similar strategy to Long Ranger for identifying SVs, but it performs local assembly on barcoded reads to test high-resolution complex SVs. Recently, a new SV calling software was presented, called LEVIATHAN [96], which can detect SVs in highly fragmented and heterozygous genomes using similar methods.

Aquila [97] is a novel method for identifying genetic variations in personal genomes by utilizing Linked Read data and a reference sequence. An extension of Aquila specifically designed for stLFR data, Aquila_stLFR [98], has been utilized to identify SVs from diploid assembly with higher accuracy compared to other methods such as GROC-SVs [44] and NAIBR [99]. AquilaSV [22] uses region-specific diploid assembly from Linked Read data to identify and analyze SVs.

A “split molecule” approach has also been proven successful, by identifying molecules which are linked together, but aligned to disjoint parts of the genome. VALOR [100] has been developed to discover large genomic inversions from Linked Read data by an algorithm based on this “split molecule” signature and read pair signature, and an improved version, VALOR2 [101], can identify not only inversions but also other complex SVs involved in segmental duplications, translocations, and deletions. LinkedSV [102] also uses “split molecule” methods to simultaneously integrate barcode overlapping and enriched fragment endpoints to identify large SVs.

NAIBR [99] identifies SVs by combining the “split molcule” approach with a probabilistic model, and similarly, Xia et al. [103] developed the ZoomX tool using probabilistic models. SV signals would be represented in Linked Reads, meaning that ZoomX can detect novel genomic junctions and hence identify large rearrangements (> 200 kb).

The Linked-Read Toolkit (LRTK) is designed to be a versatile toolkit that provides specific functions to identify genomic variation by taking advantage of the unique features of different Linked Read platforms [104].

## Conclusion

In this review, we discussed the methodologies and applications of long-range, non-local sequencing technologies, focussing on the Hi-C technology through the Arima Genomics V1 and V2 platforms as well as the Linked Read platforms of 10X, haplotagging, and TELL-seq. By assessing the published literature, we found that Hi-C has been widely used in genome scaffolding to assemble the genome at a chromosomal level, using a wide variety of algorithmic approaches. Hi-C technology has also been used for assembly curation and evaluation. In addition, despite some attempts, Hi-C remains used for SV detection due to uniformed insert length distributions. The various Linked Read platforms have been demonstrated to enhance the value of short reads for genome assembly and, in contrast to the Hi-C platforms, widely used for improved SV detection.

We also introduced metrics to assess the quality of the sequencing data produced by these platforms, and briefly demonstrated that these metrics provided a robust insight into the ability of the platforms to provide useful genomic information to researchers’ finding. For example, the Arima Genomics V2 platform produces significantly higher quality data than the V1 platform. Apart from Arima Genomics Hi-C protocol, there are a number of Hi-C products currently available in the market for selection: such as Omni-C from Cantata Bio (https://cantatabio.com/dovetail-genomics/products/omni-c/), Phase Genomics Hi-C (https://phasegenomics.com), and EpiTect Hi-C from QIAGEN (https://www.qiagen.com/gb/products/discovery-translational-research/epigenetics/epitect-hi-c-kit). Each technology has its own features, and the users may evaluate the data quality based on the matrics proposed here. Interestingly, there is also a long-read platform for selection: Pore-C from ONT [105]. The long-sequencing and long-range nanopore reads may play an important role in generating higher-order structural information.

From our analysis of the existing literature as well as our quality metrics, we found that long-range protocols, including Hi-C and Linked Read methods, significantly improve the quality of genome assembly and enhance the detection of genomic SVs. As NGS technologies and associated software pipelines continue to develop further, these technologies will continue to move from strength to strength.

We have emphasized throughout this review the distinction between true long-read platforms and long-range technologies which employ genome partitioning and barcoding to cluster reads into groups, providing much needed long-range information with only a modest increase in cost over standard short-read sequencing. Whilst the development of long-read technologies would initially seem to make the short-read-based technologies discussed here less attractive to researchers, we have demonstrated robustly that non-local information can help supplement long-read endeavours, and avoid some of the drawbacks of these emerging technologies, such that a combined long-read/long-range approach remains a cost-effective strategy for complex genome and pan-genome assemblies, population genetics, and high-resolution analysis of complex traits.

Hi-C reads have been widely used for scaffolding and phasing while Linked Reads are no longer involved in genome assembly. The strength of Linked Reads is that researchers can construct long haplotypes cheaply at low coverage, a feature which makes it suitable for population-scale studies with hundred samples at one time. Most importantly, DNA input can be as low as 1.5 ng while the evenness of coverage is good. It should be noted that long-read platforms are also developing low-input modes for applications, such as PacBio’s low and ultra-low input modes. However, the minimum input for ultra-low modes is 5 ng and the evenness of base coverage is not as good as that of normal input library. In DToL, three libraries of normal, low, and ultra-low inputs are constructed for the species which have difficulties in extracting enough DNA. The reads from these different libraries are mixed together for genome assembly with an aim to minimize the amount of DNA inputs in order to produce quality assemblies. For population-scale studies at low coverage, this method is not applicable due to high costs. Therefore, there is a room for Linked Reads to play.

We do note that, as of June 30, 2020, 10X Genomics discontinued the sale of Chromium Genome and Exome product lines — the most prominent Linked Read platform, on which a significant portion of the literature was focused. Various alternatives have been suggested, such as Sage Science’s TELL-seq, MGI stLFR, and haplotagging discussed in this review. Whilst we found that haplotagging data are in some cases of a higher quality than 10X, haplotagging beads are not (yet) commercially available, being obtainable from the Chan Lab at the Friedrich Miescher Laboratory of the Max Planck Society in Tuebingen only via academic collaboration. Commercial supply of these reagents could make haplotagging a powerful tool, as the beads are potentially inexpensive. For future Linked Read platforms, maintaining the lower level of DNA input is the key in order for researchers to perform population-scale studies. At the same time, reasonable molecular length and low costs for library production are also essential for a wide range of applications.

## Supplementary Material

qzae048_Supplementary_Data

## Data Availability

Hi-C reads were sequenced and provided by Arima Genomics while all the haplotagging datasets were produced at the Wellcome Sanger Institute as part of DToL project. The datasets have been submitted to NCBI (BioProject: PRJEB58366). These newly sequenced reads have also been deposited in the BioProject [106] at the National Genomics Data Center, Beijing Institute of Genomics, Chinese Academy of Sciences / China National Center for Bioinformation (BioProject: PRJCA020117), and are publicly accessible at https://ngdc.cncb.ac.cn/bioproject/.

## CRediT author statement

**Libo Jiang:** Conceptualization, Writing – original draft. **Michael A. Quail:** Conceptualization, Resources. **Jack Fraser-Govil:** Writing – review & editing, Methodology, Formal analysis. **Haipeng Wang:** Visualization. **Xuequn Shi:** Visualization. **Karen Oliver:** Resources. **Esther Mellado Gomez:** Resources. **Fengtang Yang:** Supervision. **Zemin Ning:** Conceptualization, Software, Writing – review & editing. All authors have read and approved the final manuscript.
