# Supplementary material for: The Bioinformatic Applications of Hi-C and Linked Reads
Source: Genomics Proteomics Bioinformatics. 2024 Jun 21;22(4):qzae048. doi: 10.1093/gpbjnl/qzae048 (PMC11580686; doi:10.1093/gpbjnl/qzae048)
Supplement: qzae048_Supplementary_Data [file qzae048_supplementary_data.zip › Table-S1.docx]

| **Dataset** | **Platform** | **Genome length** | **Fragments-per-barcode** | **1% collision size (kb)** | **Mean fragment length (kb)** | **Collision frequency (%)** |
| --- | --- | --- | --- | --- | --- | --- |
| Human-NA12878 | 10x | 6.3 Gb | 10 | 2100 | 59.2 | 0.03 |
| Hummingbird | 10x | 1.8 Gb | 10 | 580 | 44.6 | 0.08 |
| Human | Haplotagging | 6.3 Gb | 5 | 4200 | 56.2 | 0.01 |
| Rat | Haplotagging | 5.5 Gb | 5 | 3700 | 57.2 | 0.02 |
| Oak | Haplotagging | 1.4 Gb | 5 | 970 | 38.5 | 0.04 |

**Table S1 Collision frequency analysis of the Linked Read platforms**
