# Supplementary material for: The Bioinformatic Applications of Hi-C and Linked Reads
Source: Genomics Proteomics Bioinformatics. 2024 Jun 21;22(4):qzae048. doi: 10.1093/gpbjnl/qzae048 (PMC11580686; doi:10.1093/gpbjnl/qzae048)
Supplement: qzae048_Supplementary_Data [file qzae048_supplementary_data.zip › Supplementary material captions.docx]

­**Supplementary materials**

# File S1 Evenness metric

# File S2 Instructions on running assembly pipelines

**Figure S1** **Demonstration of statistical properties of evenness metric**

An example of how generating distributions$f(\lambda)$ (top) result in different Poly-Poisson distributions (bottom). All distributions are chosen to have the same mean as the black curve (the Illumina human data from Figure 3), but with other parameters chosen for demonstration purposes rather than to provide a good fit to the data. The multimodal models demonstrate that even though the Poisson distribution is monomodal, suitable generating functions can generate multimodel Poly-Poisson distributions.

**Figure S2** **Analysis of barcode collision rates**

Top is various distributions of the number of fragments of HMW-DNA which share a barcode. The blue and orange curves are designed to approximate Haplotagging, whilst the green curve demonstrates 10x. Bottom is the probability of barcode “collisions” which results as a function of the length of the fragment, assuming a diploid genome length of 6.3 Gb. Solid lines demonstrate direct collisions, where overlapping fragments share the same barcode, whilst the dashed lines show “buffered collisions”, where the shared-barcode fragments do not overlap, but are too close together for reads to be unambiguously assigned to one or the other.

**Table S1 Collision frequency analysis of the Linked Reads platforms**
