# Supplementary material for: The Bioinformatic Applications of Hi-C and Linked Reads
Source: Genomics Proteomics Bioinformatics. 2024 Jun 21;22(4):qzae048. doi: 10.1093/gpbjnl/qzae048 (PMC11580686; doi:10.1093/gpbjnl/qzae048)
Supplement: qzae048_Supplementary_Data [file qzae048_supplementary_data.zip › FileS1.docx]

**File S1 Evenness metric**

In the ideal case, the sampling of the genome would be perfectly uniform, such that every base was covered exactly the same number of times. Since this is practically impossible, we would instead prefer that every base had the same chance of being covered, and allowing for some statistical noise. If we model the sequencing process as one which samples each base of the genome at a mean rate $\lambda$, which is independent of the sampling rate of other bases, then the probability that a given base enters the library $k$ times (*i.e.*, has a coverage of $k$) is:

$$p\left( k | \lambda\right)= \frac{\lambda^{k}e^{-\lambda}}{k!}= \mathcal{P}(k|\lambda)$$

( 4 )

This is the standard Poisson distribution, and would be the result of a perfectly even sampling of the genome. If, however, there is not a single value of $\lambda$, but multiple different values, such that the probability of a given value of $\lambda$ is given by the distribution function $f\left( \lambda\right)$, then the probability of finding a coverage value of $k$ is given by a Poly-Poisson distribution, $p\left( k | f \right)$ such that:

$$p\left( k|f \right)= \int_{0}^{\infty} f\left( \lambda\right)\frac{\lambda^{k}e^{-\lambda}}{k!} \text{d}\lambda.$$

( 5 )

The integral is carried out over the full support of the parameter $\lambda$, that being the half-infinite interval. We note that if $f$ is a normalized distribution function on this interval, then the total probability still obeys:

$$\sum_{k=0}^{\infty} p\left( k | f \right)=\int_{0}^{\infty} f\left( \lambda\right)\left( \sum_{k=0}^{\infty} \frac{\lambda^{k}e^{-\lambda}}{k!} \right) \text{d}\lambda$$

$$= \int_{0}^{\infty} f\left( \lambda\right)\text{d}\lambda=1$$

( 6 )

The mean and the variance of a Poly-Poisson distribution are found from:

$$\left\langle k \right\rangle= \sum_{k=0}^{\infty} k p\left( k | f \right)= \int_{0}^{\infty} \lambda f\left( \lambda\right)\text{d}\lambda$$

( 7 )

$$\text{Var}\left( k \right)\text{ = }\left( \sum_{k=0}^{\infty} k^{2} p\left( k | f \right) \right)\text{-}\left\langle k \right\rangle^{2}\text{ }= \int_{0}^{\infty} \lambda^{2}f\left( \lambda\right)\text{d}\lambda+ \left\langle k \right\rangle\text{- }\left\langle k \right\rangle^{2}$$

( 8 )

We note that the dimensional conflict of $\left\langle k \right\rangle$ and $\left\langle k \right\rangle^{2}$ appearing in linear combinations is not a problem since the Poisson distribution inherently only deals with dimensionless “counts”. Writing the results above in terms of the variance and mean of $f$, we find that:

$$\left\langle\text{coverage} \right\rangle= \left\langle f \right\rangle$$

$$\text{Var}\text{(coverage) = }\text{Var}\left( f \right)+ \left\langle f \right\rangle$$

( 9 )

Previous works have set $f\left( \lambda\right)$ equal to the Gamma distribution, in which case $p(k|f)$ is equal to the negative binomial distribution. However, we note that there is no particular need to assign a functional form to $f$, since all we are interested in is the dispersion of this relationship around the mean. The index of dispersion is given by:

$$D(f)=\frac{\text{Var(f)}}{\left\langle f \right\rangle}$$

( 10 )

Hence:

$$D= \frac{\text{Var(coverage) - }\left\langle\text{coverage} \right\rangle}{\text{Var}\text{(coverage) }}$$

( 11 )

We reiterate that this is a measure of the dispersion of $f$ around its mean, and is hence a measure of how Poisson-like the data is: zero indicates that the data is perfectly Poisson like, whilst larger values indicate that there are a significant number of processes altering how the genome is sampled. We therefore use this quantity as a metric of the unevenness of the coverage of the genome, $\mathcal{U}=D$, with smaller values being indicative of a more even coverage.
