# Supplementary material for: The Bioinformatic Applications of Hi-C and Linked Reads
Source: Genomics Proteomics Bioinformatics. 2024 Jun 21;22(4):qzae048. doi: 10.1093/gpbjnl/qzae048 (PMC11580686; doi:10.1093/gpbjnl/qzae048)
Supplement: qzae048_Supplementary_Data [file qzae048_supplementary_data.zip › FileS2.docx]

# **File S2 Instructions on running assembly pipelines**

**Software packages**

*scaffHiC*

<https://github.com/wtsi-hpag/scaffHiC>

Note: scaffHiC contains PretextMap and we here use scaffHiC to process data and generate high-throughput chromosome conformation capture (Hi-C) maps as well as length distributions. We did not use it for scaffolding as YaHS is noteably better in genome scaffolding.

*PretextView*

https://github.com/wtsi-hpag/PretextView

*Purge_Dups*

https://github.com/dfguan/purge_dups

*YaHS*

https://github.com/c-zhou/yahs

*samtools*

https://github.com/samtools/

**Produce sorted bam file AJ.bam**

$/src/scaff-bin/bwa-mem2 mem -t 54 -5SPM GRCH38.fasta $HiC/arima/human/QC/GM24385.AJ.R1.fastq.gz $HiC/arima/human/QC/GM24385.AJ.R2.fastq.gz > align-AJ.sam

samtools view -@ 50 -bS align-AJ.sam > Sorted_names.bam

samtools fixmate -@ 50 -m Sorted_names.bam Fixmate.bam > try.out

samtools sort -@ 50 -o Sorted.bam Fixmate.bam > try.out

rm -rf align-AJ.sam Sorted_names.bam Fixmate.bam

samtools markdup -@ 50 -r -s Sorted.bam Dupmarked.bam > try.out

mv Dupmarked.bam AJ.bam

**Coverage analysis**

samtools depth Sorted.bam | egrep _0 | awk '($2%100==0){print $0}' > depth.dat

sort -n -k 3 depth.dat | awk '{print $1,$3}' > depth-raw.dat

$/src/scaff-bin/distribution_hic-coverage depth-raw.dat | awk '{print $2,$3}' > depth-freq.dat

**Hi-C contact map**

$/src/scaffhic -nodes 54 -depth 50 -score 200 -map arima-AJ.map -plot arima-AJ.png -length 500000 -file 0 -fq1 $HiC/arima/human/QC/GM24385.AJ.R1.fastq.gz -fq2 $HiC/arima/human/QC/GM24385.AJ.R2.fastq.gz GRCH38.fasta aj-arima.fasta > try.out

Here we obtained arima-AJ.map and arima-AJ.png. You may use PretextView to view the Hi-C map: https://github.com/wtsi-hpag/PretextView

**Genome assembly**

*Contigs*

~zn1/src/hifiasm/hifiasm -o hg002-hifiasm -t 80 HG002-HiFi-all.fastq.gz > try.out

egrep "^S" hg002-hifiasm.p_ctg.gfa | awk '{print ">"$2"\n"$3}' > hg002-hifiasm.fasta

*Purge_Dups*

$src/minimap2/minimap2-2.17_x64-linux/minimap2 -t 30 -xmap-pb hg002-hifiasm.fasta HG002-HiFi-all.fastq.gz | gzip -c - > align.paf.gz

$src/purge_dups/bin/pbcstat align.paf.gz

$src/purge_dups/bin/calcuts PB.stat > cutoffs

$src/purge_dups/bin/split_fa hg002-hifiasm.fasta > Human.split

$src/minimap2/minimap2-2.17_x64-linux/minimap2 -t 20 -xasm5 -DP Human.split Human.split | gzip -c - > split.self.paf.gz

$src/purge_dups/bin/purge_dups -2 -T cutoffs -c PB.base.cov split.self.paf.gz > dups.bed

$src/purge_dups/bin/get_seqs dups.bed hg002-hifiasm.fasta > purged.fa 2> hap.fa

*Scaffolding*

$/src/scaff-bin/bwa-mem2 mem -t 54 -5SPM purged.fa $HiC/arima/human/QC/GM24385.AJ.R1.fastq.gz $HiC/arima/human/QC/GM24385.AJ.R2.fastq.gz > align-purge.sam

samtools view -@ 50 -bS align-purge.sam > Sorted_names.bam

samtools fixmate -@ 50 -m Sorted_names.bam Fixmate.bam > try.out

samtools sort -@ 50 -o Sorted.bam Fixmate.bam > try.out

rm -rf align-AJ.sam Sorted_names.bam Fixmate.bam

samtools markdup -@ 50 -r -s Sorted.bam Dupmarked.bam > try.out

mv Dupmarked.bam AJ-scaff.bam

~zn1/src/yahs/yahs -o HG002-yahs.fa purged.fa AJ-scaff.bam > try.out

*Hi-C map for scaffolded assembly*

$/src/scaffhic -nodes 54 -depth 50 -score 200 -map yahs-final-AJ.map -plot yahs-final-AJ.png -length 500000 -file 0 -fq1 $HiC/arima/human/QC/jz1/GM24385.AJ.R1.fastq.gz -fq2 $HiC/arima/human/QC/jz1/GM24385.AJ.R2.fastq.gz HG002-yahs.fa arima-AJ.fasta > try.out

Here we have yahs-final-AJ.map and yahs-final-AJ.png.
