# Supplementary figures and images for: The Bioinformatic Applications of Hi-C and Linked Reads
Source: Genomics Proteomics Bioinformatics. 2024 Jun 21;22(4):qzae048. doi: 10.1093/gpbjnl/qzae048 (PMC11580686; doi:10.1093/gpbjnl/qzae048)

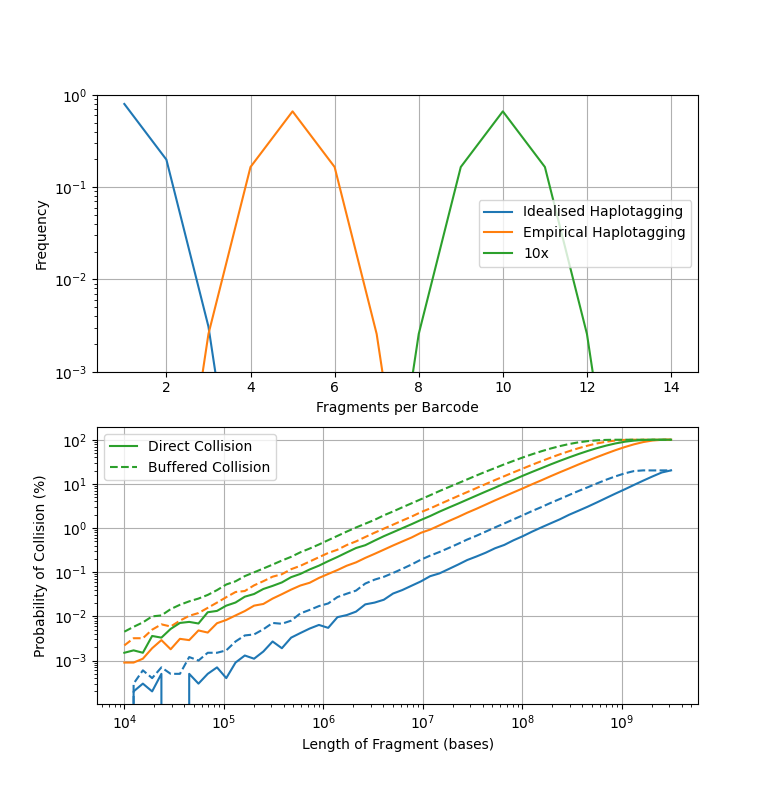

Supplement: qzae048_Supplementary_Data [file qzae048_supplementary_data.zip › FigureS2.docx]
